# Surgical Information Available on the Box of Cardiac Valve Prostheses Remains Inadequate

**DOI:** 10.1093/icvts/ivag077

**Published:** 2026-03-19

**Authors:** Astrid Gerritje Maria van Boxtel, Tjark Ebels

**Affiliations:** Department of Cardio-Thoracic Surgery, University Medical Centre Groningen, 9732GZ Groningen, Netherlands; Department of Cardio-Thoracic Surgery, University Medical Centre Groningen, 9732GZ Groningen, Netherlands

The boxes of surgical cardiac valve prostheses are routinely known by cardiac surgical operative nurses, in particular, the ease or difficulty with which to open them, but they are largely overlooked by most cardiac surgeons. This lack of interest is regrettable since these boxes could and should provide essential and useful information. However, on most of the boxes, there is still ample room for improvement to inform surgeons optimally about the metrics of the prosthetic valve they contain. In particular, as during the operation, this is the last chance prior to having actually bought the prosthesis, and to avoid problems like patient prosthesis mismatch (PPM).

Unknown to almost all cardiac surgeons and cardiologists alike, the requirements for accurate and comprehensive information available on the box of cardiac valve prostheses is codified by ISO, the International Standards Organization in Geneva, in a document called a Standard with a capital S. ISO and some other standards organizations codify almost all technical and procedural processes in our life. The obvious goal is to offer uniformity and adequacy of that very information. Dedicated ISO working groups are populated mostly by employees of manufacturers and a few from governments, “experts,” and the odd medical doctor. The manufacturers are usually in the majority, and significantly, there is no participation from patients, the ultimate beneficiaries of uniform and adequate information on the box. However, recent literature^1^^,^^2^ has shown the information to be not uniform at all, nor adequate, let alone correct. Apparently, well-intended ISO procedures have proven not to lead to outright uniformity that enables surgeons, regulating and enforcing authorities to protect patients’ interests. As surgeons, in particular, should protect patients’ interests, we should be aware of the confusion about the metrics of prostheses and how to deal with this knowledge.

Since July 1975, there is a Standard by ISO on cardiac valve prostheses. The current Standard describing the requirements for surgical heart valves is the ISO Standard 5840 and was released in 1984. Since then, there have been 5 updated versions (1984, 1989, 1996, 2005, 2015, 2021). The most current version is the 2021 Standard,^3^ followed by an ISO publicly available specification (ISO PAS) in 2023^4^ and amendments on this ISO PAS in 2025.^5^

It takes a total of seven documents (ISO 5840–1; 2021, ISO 5840–2; 2021, 2023–2 and 2025, ISO 15223–1:2016, ISO 14630:2012, ISO 11607) and a lot of exploration of many references and replacements within and among documents, in order to clarify which information should be provided on the packaging boxes. All these ISO documents have to be paid for dearly to purchase them privately. ISO Standards numbers of pages increased exponentially over time. All this additional information should provide for clarification but paradoxically creates the opposite effect and causes one to lose sight of the bigger picture.

**Figure 1.**
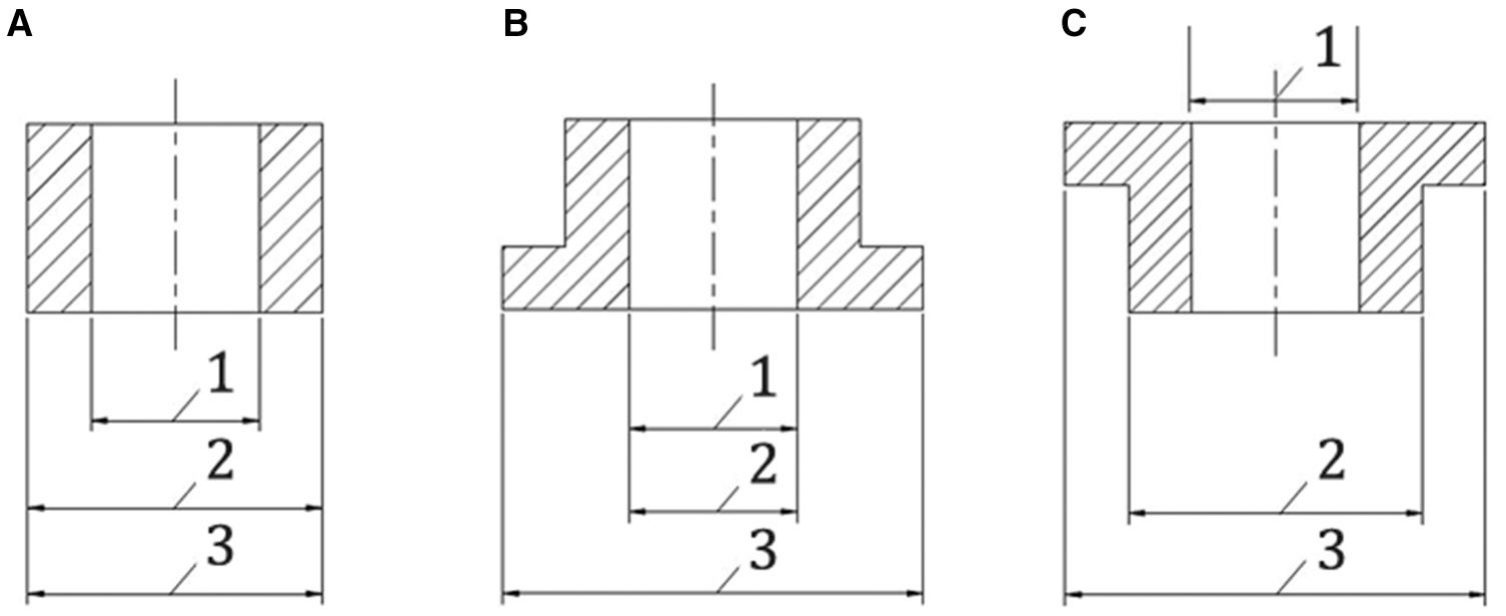
Designation of Dimensions of Surgical Heart Valve Substitute Sewing Ring Configurations

Examining all those documents results in the following list of metrics which should be printed on the outside of the packaging box of surgical heart valve prostheses:

- intended valve to be replaced;
- intended position in relation to the annulus;*
- inflow orifice diameter;
- effective orifice diameter;
- valve housing external diameter;
- external sewing ring diameter.

* since the 2023 ISO PAS, “*intended position in relation to the annulus*” is not designated as information to be put on the outside of the packaging box. However, in our opinion, this is crucial information since it determines whether the prosthesis is labelled as intra-annular or as supra-annular. The essential difference being that the labelled diameter = patient annulus diameter = valve housing external diameter, or as a supra-annular valve in which labelled diameter = patient annulus diameter = inflow orifice diameter, which is explained visually in Figure 1.

On top of this confusion, ISO dictates that the effective orifice diameter (ID effective) is on the box. The ID effective is not a metric, but a virtual diameter derived from hydrodynamic performance data measured with a standard validated procedure. A crucial metric, because it describes the haemodynamic diameter “between the leaflets,” and directly correlates with the amount of obstruction, if any, to blood that passes through the prosthesis, an important indication for most valve replacements. However, currently, none of the manufacturers puts the ID effective on their boxes. The geometric inflow orifice diameter was measured in our study in 2023,^1^ using a conical gauge. This metric is a predictor for PPM and mortality as described by Hamilton et al.^6^ Functionally, it should be tested with a steady flow setup. There is only one publication^7^ in which some of those data are published, but these data are flawed since the test setup was not fully correct, using the same size outlet tube for all sizes of valves. So in fact, one of the most relevant diameters of the valve prostheses is unknown to most of the users. These metrics are also absent in all internet-based information on these valve prostheses.

Next to ISO, the 3 major professional associations (EACTS, STS, and AATS) have published different suggestions^8^^,^^9^ to clarify information provision necessary on the box. Paradoxically, since those proposals are not consistent with the ISO Standards, they add to the confusion instead of simplification.

It would be much wiser if the professional organizations would participate in the ISO working group, for which ISO is open. This would provide for a setting promoting uniformity and thereby clarification. This is not only the case for this ISO working group but also for other relevant Standards in cardio-thoracic surgery such as extracorporeal circulation.

There could be an inhibition to publish this critical note about the process of valve labelling, because scientific journals are often owned by professional associations and editors could feel hesitant to infringe upon in the financial interest of the owners. Due to (financial) dependency they might feel hesitant to criticize manufacturers. Fortunately, this journal shows that despite a financial dependency, it is ready to publish all sides of this story. In this way, it even contributes to bringing these issues out into the open.

This editorial describes the efforts to clarify the information on packaging boxes. Despite these efforts to simplify and correct information provision, there are hardly any improvements. We revealed the challenges and conflicts of interests which play a role in this. We sincerely hope that we could spark the discussion and initiate and contribute to a change for the better.
